# Computational Methods for Single-cell DNA Methylome Analysis

**DOI:** 10.1016/j.gpb.2022.05.007

**Published:** 2022-06-17

**Authors:** Waleed Iqbal, Wanding Zhou

**Affiliations:** 1 Center for Computational and Genomic Medicine, Children’s Hospital of Philadelphia, Philadelphia, PA 19104, USA; 2 Department of Pathology and Laboratory Medicine, University of Pennsylvania, Philadelphia, PA 19104, USA

**Keywords:** Single-cell genomics, Bioinformatics, DNA methylation, Computational tool, Epigenetics

## Abstract

Dissecting intercellular epigenetic differences is key to understanding tissue heterogeneity. Recent advances in single-cell DNA methylome profiling have presented opportunities to resolve this heterogeneity at the maximum resolution. While these advances enable us to explore frontiers of chromatin biology and better understand cell lineage relationships, they pose new challenges in data processing and interpretation. This review surveys the current state of **computational tools** developed for single-cell DNA methylome data analysis. We discuss critical components of single-cell DNA methylome data analysis, including data preprocessing, quality control, imputation, dimensionality reduction, cell clustering, supervised cell annotation, cell lineage reconstruction, gene activity scoring, and integration with transcriptome data. We also highlight unique aspects of single-cell DNA methylome data analysis and discuss how techniques common to other single-cell omics data analyses can be adapted to analyze DNA methylomes. Finally, we discuss existing challenges and opportunities for future development.

## Introduction

DNA methylation typically refers to the methylation of the 5-carbon of the cytosine base. It is one of the most classic epigenetic modifications in higher-order eukaryotes [1], [2], [3], [4]. DNA methylation consolidates epigenetic states over cell replication and is extensively implicated in many biological activities: transcriptional regulation [5], [6], [7], genomic imprinting [8], [9], [10], [11], X-chromosome inactivation [12], [13], transposable element suppression [14], [15], [16], [17], cell differentiation [18], [19], [20], and tissue and organismal development [21], [22], [23], [24], [25]. It has also been extensively studied and applied to track aging [26], [27] and various forms of human diseases [28], [29], [30], [31], [32], [33], [34].

Many non-recurrent cellular alterations with phenotypic manifestation are inheritable over mitosis and coded into the epigenome. As a classic epigenetic mark, the DNA methylome encodes rich information that delineates the state of a cell, including its developmental lineage, cell cycle stage, transcriptional activity, mitotic age, and proliferation potential. In this review, the term “methylome” will refer to the genome-wide distribution of 5-methylcytosine in DNA. Other forms of DNA and protein methylation (*e.g.*, histone methylation) will be specified explicitly. The most effective strategy for capturing this cell-to-cell diversity is to profile the methylomes of single cells. Like other single-cell omics assays, the single-cell DNA methylome assay has been actively developed with a rapid surge in data volume and variety over the past decade. Table 1 highlights the limitation of bulk methylome analyses compared to their single-cell counterparts.

**Table 1:** **Comparison of typical analyses performed on single-cell and bulk methylome sequencing data**

**Analysis**	**Bulk tissue**	**Single cell**
Tissue- or cell-specific epigenome	Lineage relationship of the major constituent cell states of tissues	Differentiation trajectory of single cells and cell states
Cell composition analysis	Top-down cell composition analysis of convoluted tissue signals	Bottom-up cell composition analysis focusing on rare cell types
DNA copy number annotation	Weighted average DNA copy number from the whole cell population	DNA copy numbers of each cell and its heterogeneity
DNA copy number on sex chromosomes	Sex inference and sex chromosome abnormality of the whole individual	Sex chromosome epigenetic mosaicisms across cell types
Primary tumor epigenetic alteration	Focus on tumor *vs*. normal epigenetic differences such as global hypomethylation	Distinguish cell-autonomous epigenetic changes in individual tumor cells
Tumor cell evolution history	Compare the epigenome of tumor at multiple sites (*e.g.*, primary *vs*. metastatic)	Resolve clonal evolution history of tumor cells
Cell cycle	Compute the fraction of cells at different cell cycle stages	Determine cell cycle stage of individual cells
Epigenome–transcriptome association	Epigenome–transcriptome associations influenced by cell type variation	Epigenome–transcriptome associations across cells of the same cell type

Compared to single-cell transcriptomic data, which carry information on the transcriptional state, DNA methylome data carry information on mechanisms of gene expression regulation, such as the involvement of specific *cis*-regulatory elements and their interactions. Although this genome-wide coverage enables detecting gene regulatory differences overlooked in single-cell transcriptomics, single-cell methylome data contain signals from hundreds of thousands to millions of CpGs in the genome, representing much higher dimensional data. In other words, the data sparsity challenge, common to all single-cell data, is particularly prominent for single-cell DNA methylome data due to the limitation of DNA (compared to RNA) material per locus per diploid cell. In addition, sodium bisulfite conversion [35], [36], a technique commonly used in methylome profiling, causes DNA damage and further contributes to DNA loss and data sparsity.

DNA methylation data have an uneven genomic representation with a complex spatial correlation pattern. CpG dinucleotides, where most DNA methylation readings are collected, are unevenly distributed in the genome (non-CpG or CpH methylation is only found in some tissue and cell types such as neurons and embryonic stem cells). Different genomic features have different CpG densities. Late-replicating lamina-attached DNA is CpG-sparse [37], while CpG island DNA retains higher CpG density [38]. The CpG island lengths at different gene promoters are on a continuous spectrum. DNA methylation of neighboring CpGs is intrinsically correlated with different genomic scales depending on the underlying cellular and biochemical processes. The determination of DNA methylation at *cis*-regulatory elements [18] and promoters [39] is typically focal, and loss of methylation is often indicative of transcriptional machinery binding. However, some transcription factors (TFs) preferentially bind methylated sites [40], [41]. In contrast, loss of methylation at partially methylated domains (PMDs) due to extensive cell division occurs on mega-base scales [42], [43]. This complex grammar of DNA methylation determination mandates consideration of CpG distribution and its correlative structure at multiple genomic scales during bulk-tissue and single-cell DNA methylome analyses [44].

Computational and statistical techniques are emerging for single-cell methylome analysis, though they are not catching up with the development of informatics for single-cell RNA sequencing (scRNA-seq) and single-cell assay for transposase-accessible chromatin sequencing (scATAC-seq). This lag is partly due to the challenges and complexities mentioned above. This review surveys the current computational methods developed for single-cell DNA methylome analysis (Table 2). As some methods share principles common to the analyses of bulk-tissue DNA methylome data and other single-cell omics data, we will focus on the unique challenges of single-cell DNA methylome analysis.

**Table 2:** **Overview of analytical features of single-cell methylome analysis tools**

	**General information**		**Input data**			**Data processing**					**Cell state annotation**			**Modeling**		**Multi-omics**			**Data presentation**	
**Name**	**Programming language**	**Benchmarked dataset**	**Epigenome**	**scRNA-seq integration**	**Data format**	**Filtering**	**Normalization**	**Imputation**	**Feature matrix**	**Feature engineering**	**Motif analysis**	**Cell typing**	**Cell trajectory**	**Clustering**	**Differential methylation**	**Label transfer**	**Gene activity scoring**	**Cell space**	**Visualization**	**Ref.**
BPRmeth	R	scNMT	M		MC	F+V		+	RC	SVLR				+	+		+		+	[134]
DeepCpG	P	scBS-seq scRRBS	M		MC			+	RC	CNN	+			+	+				+	[139]
MELISSA	R	scM&T-seq scBS-seq	M		MC		+	+	RC	BI				+					+	[143]
Epiclomal	P+R	scBS-seq scRRBS scWGBS	M		MC	F+V	+	+	RC	BI				+						[144]
MAPLE	R	snmC-seq scM&T-seq scNMT-seq	M	+	AB					ENS						S	+	DS*		[211]
MethylStar	P+R+Sh	scBS-seq	M		FQ			MI												[141]
scMET	R	snmC-seq scNMT-seq	M		MC			+	RC	BI					+					[189]
EpiScanpy	P	snmC-seq	M/A		MC	F+V+D	+	+	RC	NG			+	+	+		A		+	[138]
coupleCoC+	ML	snmC-seq	M/A	+	MG			+	GC	ITC				+			A	DS	+	[240]
ALLCools	P	snmC-seq2	M/A	+	PY	F+V+D	+		RC	+	+	+		+	+			DS	+	[66]
MATCHER	P	scM&T-seq scGEM	M/A	+	MB								+	+	+	+		DS		[218]
LIGER	R	snmC-seq	M/A	+	MG				FC	MF		+		+	+	+		DS	+	[210]
scAI	R	scM&T-seq	M/A	+	MB	F	+		FC	MF				+	+			CA	+	[228]
MOFA+	R	scM&T-seq	M/A	+	MO		+		FC	MF				+	+			CA	+	[227]

*Note*: The epigenetic input of these tools is depicted by M if they are designed solely for single-cell methylation-seq or M/A if the input can be either single-cell methylation-seq or single-cell ATAC-seq. ‘+’ indicates that the feature is supported or the functionality is through another specified software. For convenience, we have included the methylation datasets used by the original papers of these tools, for testing and training purposes; single-cell datasets not containing methylation have been omitted (scRNA-seq, scATAC-seq, or both). scRNA-seq, single-cell RNA sequencing; scATAC-seq, single-cell assay for transposase-accessible chromatin sequencing; P, Python; Sh, Shell/BASH; ML, MATLAB; M, DNA methylation; A, chromatin accessibility; MC, methylation call; AB, aligned read in BAM; FQ, FASTQ raw read; MG, methylation call by gene; PY, processed read in the YAPS MCDS format; MB, binarized methylation call; MO, MultiAssayExperiment Object; F, filtering CpGs and cells by sequencing depth or data sparsity; V, filtering CpGs by methylation variation; D, doublet detection and filtering; MI, conducted through METHImpute; RC, genomic region-by-cell matrix; GC, gene-by-cell matrix; FC, factor-by-cell matrix (factor includes shared factors and non-shared factors); SVLR, support vector linear regression; CNN, convolutional neural network; BI, Bayesian inference; ENS, ensemble machine learning (CNN + elastic net + random forest); NG, neighbor graph and graph-based clustering (Louvain, Leiden, *etc.*); ITC, information-theoretic co-clustering; MF, matrix factorization; S, conducted through Seurat; CA, integration of co-assays; DS, integration of data from different sample spaces; DS*, integration of data from different sample spaces but using co-assay data for regressor training.

We first briefly discuss DNA methylome assay technologies and data preprocessing workflows. Then we focus on common methylome analysis tasks: quality control, dimensionality reduction, cell clustering, differential methylation analysis, cell lineage analysis, motif analysis, analysis of cell-to-cell methylation concordance, and analysis of non-CpG methylations. Although single-cell DNA methylome analysis is briefly compared with data of other modalities, we refer readers to other reviews for detailed discussions of the corresponding data types: scRNA-seq [45], [46], [47], [48], scATAC-seq [49], [50], and single-cell HiC (scHiC) [51], [52]. Non-traditional uses of the DNA methylation sequencing data, such as identifying genetic mutations and copy number alterations, are also discussed. Finally, we discuss how to integrate single-cell DNA methylome data with data from other omics and how to analyze co-assay data, in which DNA methylation and other omics data were collected from the same cells.

## Single-cell DNA methylome assay technologies

**Figure 1:**
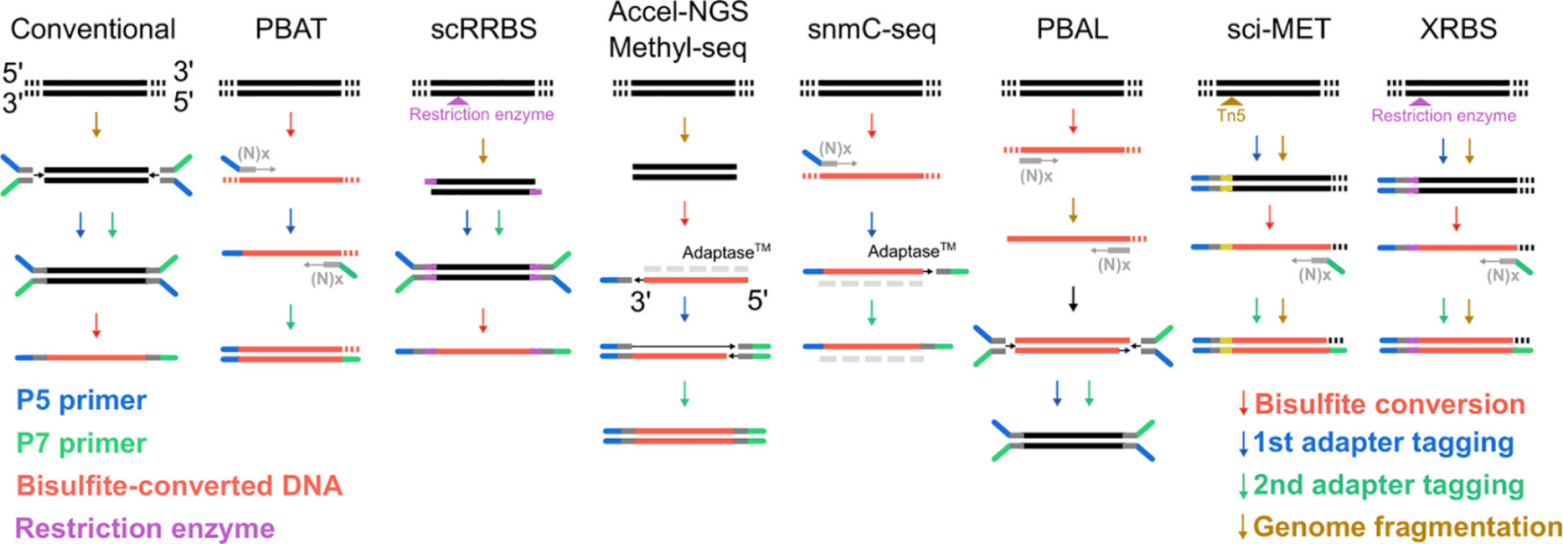
**Library preparation strategies of single-cell bisulfite sequencing assays** These assays can be differentiated based on having either bisulfite conversion (red arrow) or genome fragmentation (golden arrow) as the first step, followed by subsequent differences in adapter tagging. Blue arrows represent the first adapter tagging, and green arrows represent the second adapter tagging. Gray dashed lines denote the missing complementary strand for single-stranded DNA. Additionally, the bisulfite-converted DNA is shown in red, the insertion from the restriction enzyme is shown in purple, and the P5 and P7 primers are shown in blue and green, respectively. PBAT, post-bisulfite adaptor tagging; scRRBS, single-cell reduced representation bisulfite sequencing; Accel-NGS Methyl-seq, accel next-generation methylation sequencing; snmC-seq, single nucleus methylome sequencing; PBAL, post-bisulfite adapter ligation; sci-MET, single-cell combinatorial indexing for methylation analysis; XRBS, extended representation bisulfite sequencing.

Mainstream single-cell DNA methylome assays chemically convert cytosines to other bases depending on the cytosine’s methylation state [53]. This conversion is typically mediated by sodium bisulfite treatment [35], [36]. However, it has recently been achieved through enzymes [54] and other chemicals [55], [56], [57]. Cytosine conversion takes place on single-strand DNA and breaks the strand complementarity. Conventional library preparation procedures first attach Y-shape adapters to the double-strand DNA before bisulfite conversion. For example, the single-cell reduced representation bisulfite sequencing (scRRBS) method [58] follows this paradigm. However, bisulfite conversion damages the adapter-ligated library and causes significant DNA loss. Therefore, modern single-cell bisulfite sequencing methods attach either the second or both adapters after bisulfite conversion. An example is the post-bisulfite adapter tagging (PBAT) method [59] (Figure 1). Current single-cell methylome sequencing technologies can be classified by whether they cover the whole methylome, *e.g.*, single-cell whole-genome bisulfite sequencing (scWGBS) [60], [61], [62], [63], [64], [65], [66], or specific subsets, *e.g.*, scRRBS [58], [67], [68] and its variants [69], [70], which primarily target CpG-dense genomic regions. Differences among these methodologies are reflected in their choice of genome fragmentation method. Conventionally, fragmented library DNA was generated using DNA sonication, mechanical shearing, and random priming-based pre-amplification. Alternatively, restriction enzyme digestion [58], [69] and transposase-mediated “tagmentation” [71] allow for multiplex barcoding [67] and combinatorial barcoding [64]. These methods have higher cell number throughput and can profile hundreds to thousands of cells in one experiment. Cytosine conversion-based DNA methylome profiling methods are compatible with co-assays of other omics data that may be profiled through affinity binding, genome fragmentation, and other base conversions. Such technologies include: single-cell triple-omics sequencing (scTrio-seq) [72], [73], single-cell methylation and transcriptome sequencing (scM&T-seq) [74], single-cell nucleosome occupancy and methylome sequencing (scNOMe-seq) [75], single-cell chromatin overall omic-scale landscape sequencing (scCOOL-seq) [76], single-cell nucleosome, methylation and transcription sequencing (scNMT-seq) [77], ‘switching mechanism at the end of the 5′-end of the RNA transcript’ sequencing combined with RRBS (SmartRRBS) [68], methyltransferase treatment followed by single-molecule long-read sequencing (MeSMLR-seq) [78], single-cell methyl-HiC sequencing (scMethylHiC) [79], and single-nucleus methyl-3C sequencing (sn-m3C-seq) [80].

Other conversion-free methylation-specific restriction enzyme (MSRE)-based methods, *e.g.*, single-cell genome-wide CpG island (CGI) methylation sequencing (scCGI-seq) [81] and epigenomics and genomics of single cells analyzed by restriction (epi-gSCAR) [82], have also been developed. However, these methods do not profile the whole methylome at the base resolution. Recently, long-range sequencing [83] and imaging-based technologies have yielded equivalent single-molecule data without single-cell isolation/barcoding [84], [85], [86], [87]. These methods are promising for studying locus-specific genomic regions in single cells [54] or potentially small genomes when single reads can cover the whole chromosome or genome. We refer the readers to previous reviews to compare these single-cell methylome assays [53], [88], [89].

### Single-cell DNA methylome data preprocessing

#### Read processing and library quality control

**Figure 2:**
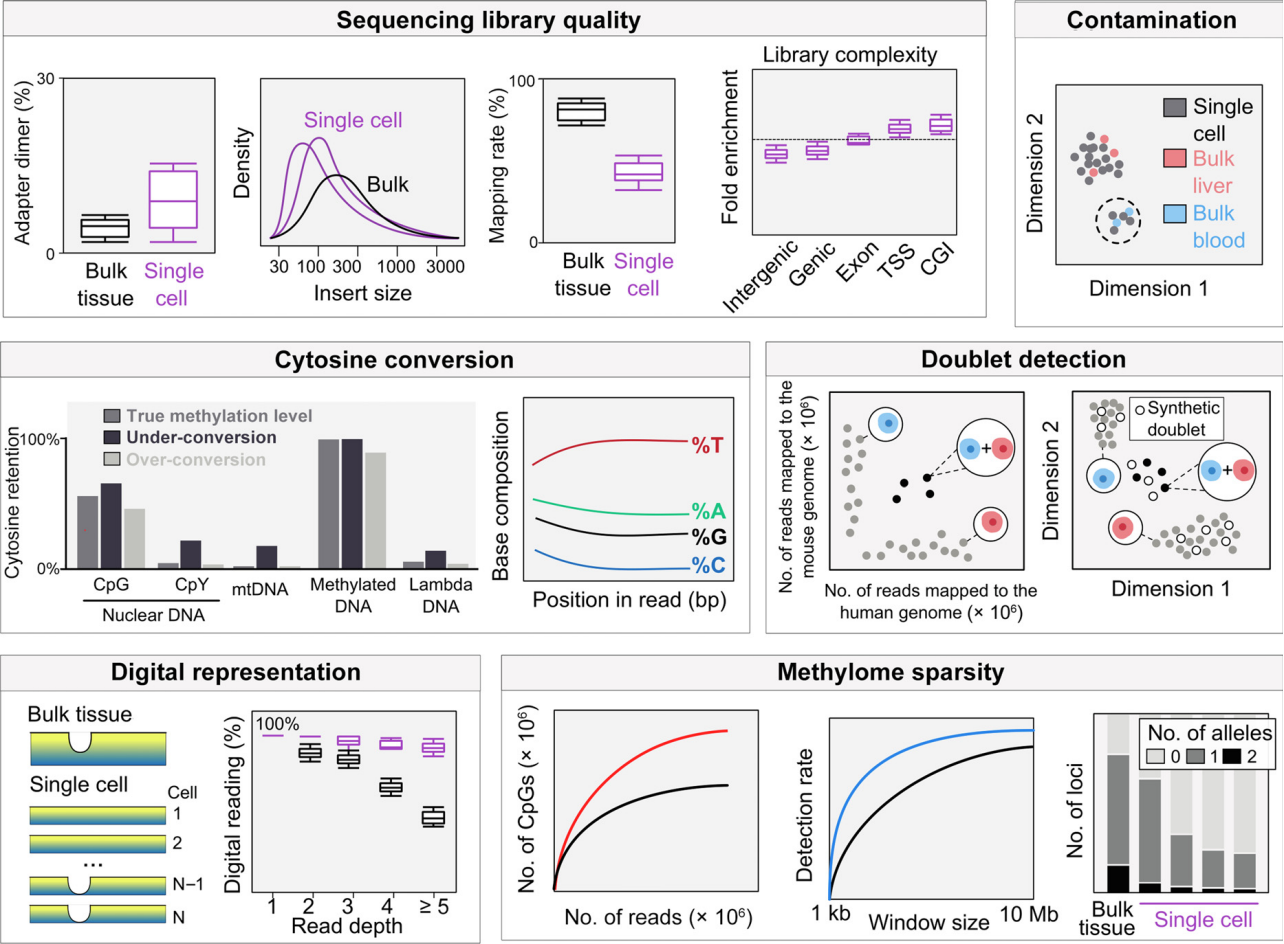
**Schematic representation of single-cell methylome data quality control** Quality control consists of sequence library assessment, sample contamination detection, doublet detection, cytosine conversion control, and filtering cells and samples based on data sparsity. Sequencing library quality: sequencing library quality can be assessed by comparing metrics such as adapter dimer (%), adapter insert size, and mapping rate (%) between bulk tissues (black) and single cells (purple); library complexity can be analyzed based on fold enrichment of different genomic regions. Contamination control: single-cell samples can be compared with their bulk counterparts to check for potential contamination. Cytosine conversion: (1) cytosine context retention [C followed by G (CpG), C followed by C or T (CpY), mitochondrial DNA (mtDNA), methylated DNA, and spike-in lambda phage DNA] and examples of expected percentages according to true methylation levels (dark gray), under-conversion (black), and over-conversion (light gray) are shown; (2) base composition percentage according to read position after bisulfite treatment [T will be present at the highest percentage (unmethylated Cs are converted to T due to bisulfite treatment, increasing total T percentage after conversion), and C will be present at the lowest percentage (only methylated Cs are retained)]. Doublet detection: (1) cross-species mixing experiment can be used to identify and determine the doublet rates in single-cell assays (*e.g.*, in a human-mouse mixing experiment, samples with high mapping rates to both human and mouse genomes are doublets); (2) clustering actual samples with synthetic doublets can also be used to filter doublets (samples that cluster close to known synthetic doublets could be marked and removed). Digital representiation: (1) comparing overall methylation patterns in bulk tissues and single cells; (2) comparing read depth and digital reading (0 or 1) percentage (%) in bulk tissues (black) and single cells (purple). Methylome sparsity: (1) comparing the number of CpGs covered as more reads are sequenced (red and black lines represent schematic examples of two different experiments or sequencing methodologies); (2) comparing the detection rate as the smoothing window size increases (blue and black lines represent schematic examples of two different experiments or sequencing methodologies); (3) single-cell data may have fewer allele-specific methylations as compared to bulk data.

Traditional read trimmers (*e.g.*, TrimGalore!) and mappers (*e.g.*, Bismark [90], BSmap [91], and BSseeker [92], [93], [94]) were first designed for bulk-tissue analysis but can be applied to single-cell data analysis with some necessary adaptations. Single-cell bisulfite sequencing library preparation often has one or multiple rounds of random priming-based pre-amplification [65]. The first sequencing adapter may tag both the bisulfite-converted strand and the daughter strand; mappers need to be aware of this change so that reads can be mapped to all four-strand types [60]. Lower mapping rates, higher percentage of adapter dimers, and smaller insert sizes are common issues seen in single-cell methylome data (Figure 2). Mappers that do not support sub-read mapping require proper trimming of leading and trailing subsequences that may be artificial due to overhang end-repair, incomplete conversion at the 5′ end, and low sequencing quality at the 3′ end [95]. To enhance mapping efficiency, mate reads in a pair may be mapped separately as single-end reads, with or without first mapping them in pairs, to accommodate potential microhomology-mediated chimerism [60], [76], [96]. The scBS-map package increases the mapping rate of such chimeric reads through local alignment [97]. Additionally, certain regions are recommended to be excluded in DNA methylation quantification due to low mappability in the bisulfite-converted genome [98].

#### Contamination control

Quality control is more relevant for single-cell methylome analysis than its bulk-tissue counterpart (Figure 2). Single-cell methylome data contain common technical confounders such as sample contamination, cytosine conversion artifacts, and the presence of doublets. To check for contamination and sample mislabeling, one can confirm donor sample identity by extracting single nucleotide polymorphism (SNP) information [99] and copy number alterations [100] from bisulfite sequencing data; cells from the same donor should have almost identical genotypes. We recommend mapping sequencing reads against potential contaminants and co-clustering single-cell data with existing bulk methylome data so that one can discriminate contaminating cells [100] (Figure 2) and evaluate the signal over noise ratio. For example, single-cell methylome data are supposed to form clusters with bulk data of similar tissue types rather than with different tissues assayed with the same technology.

#### Conversion control

Proper cytosine conversion can be challenging for DNA methylome assays since it is subject to the influence of bisulfite treatment duration [101], [102], incubation temperature [103], and choice of the polymerase for subsequent amplification [104], [105]. One could use internal or external controls to gauge proper cytosine conversion. Intrinsic controls include cytosines with assumed methylation states. Cytosines in the mitochondrial genome [65], at CpC and CpT sites [106], and at CpCpC sites [63], [66] may be used as internal controls for incomplete conversion because they often lack biological DNA methylation [107]. Although universally methylated cytosines are more difficult to find, a similar strategy can detect over-conversion. Promising targets may include CpGs at certain transposable elements whose methylation is critically maintained for cell viability [108]. Since the assumed methylation states of internal controls are subject to rare exceptions [109], one can also use external controls such as spiked-in unmethylated lambda DNA [100] or amplified and unmethylated DNA for detecting incomplete conversion, as well as M.SssI*-*treated fully methylated DNA for detecting over-conversion (Figure 2). Based on these principles, we recommend excluding incompletely converted reads (*e.g.*, by removing reads with three or more consecutive non-converted CpH cytosines [110]) or whole-cell samples (*e.g.*, requiring mCpCpC level < 0.03, mCpG level > 0.5, and mCpH level < 0.2 [66]).

#### Doublet detection

Due to the technical challenge of single-cell isolation [111], [112], the inclusion of more than one cell in a single-cell experiment can occur. The exclusion of doublets and samples with more cells is critical to single-cell methylation and ploidy analyses (*e.g.*, one based on lambda DNA spike-ins [76]). To our knowledge, no dedicated method has been developed for doublet detection for single-cell methylome data. Several strategies have been proposed and used in practice. For example, doublets typically feature an unusually high read number [64], [66] after PCR duplicate marking [90], [113]. Doublets can also have a higher number of CpGs with non-0-1 methylation levels. Most human and murine cells are diploid. Polyploidy may arise in cell types such as hepatocytes [114], cardiomyocytes [115], megakaryocytes [116], cells in the S and G2 phases, and cancer cells with somatic copy number changes [117]. A popular generic strategy developed originally for scRNA-seq (*e.g.*, DoubletFinder [118] and Scrublet [119]) and lately for scATAC-seq (*e.g.*, ArchR [120] and SnapATAC [121]) is to compare with synthetic doublets created by mixing signals from different cells. Cells that demonstrate similarity to these synthetic doublets are labeled for removal. The doublet rate of a single-cell sequencing method can be estimated using a cross-species pooling experiment where cells from different species (*e.g.*, human and mouse) are pooled before being sequenced. The single-cell data are then mapped to the two genomes separately [100]. High read mapping rates in both species indicate the presence of more than one cells [60], [122] (Figure 2).

#### Discretization of DNA methylation fractions

Since DNA methylation fractions can only be 0%, 50%, and 100% in diploid cells (Figure 2), Hui et al. considered only CpGs with 0 and 1 in methylation fraction and ignored the other CpGs [62]. One can also discretize the observed cytosine retention rate, *e.g.*, taking fractions between 0 and 0.1 to 0, between 0.9 and 1 to 1, and everything else to 0.5 [65], [123]. Smallwood et al. [65] and Argelaguet et al. [96] modeled each read as a Bernoulli random variable and inferred the methylation rate.

### Handling data sparsity

**Figure 3:**
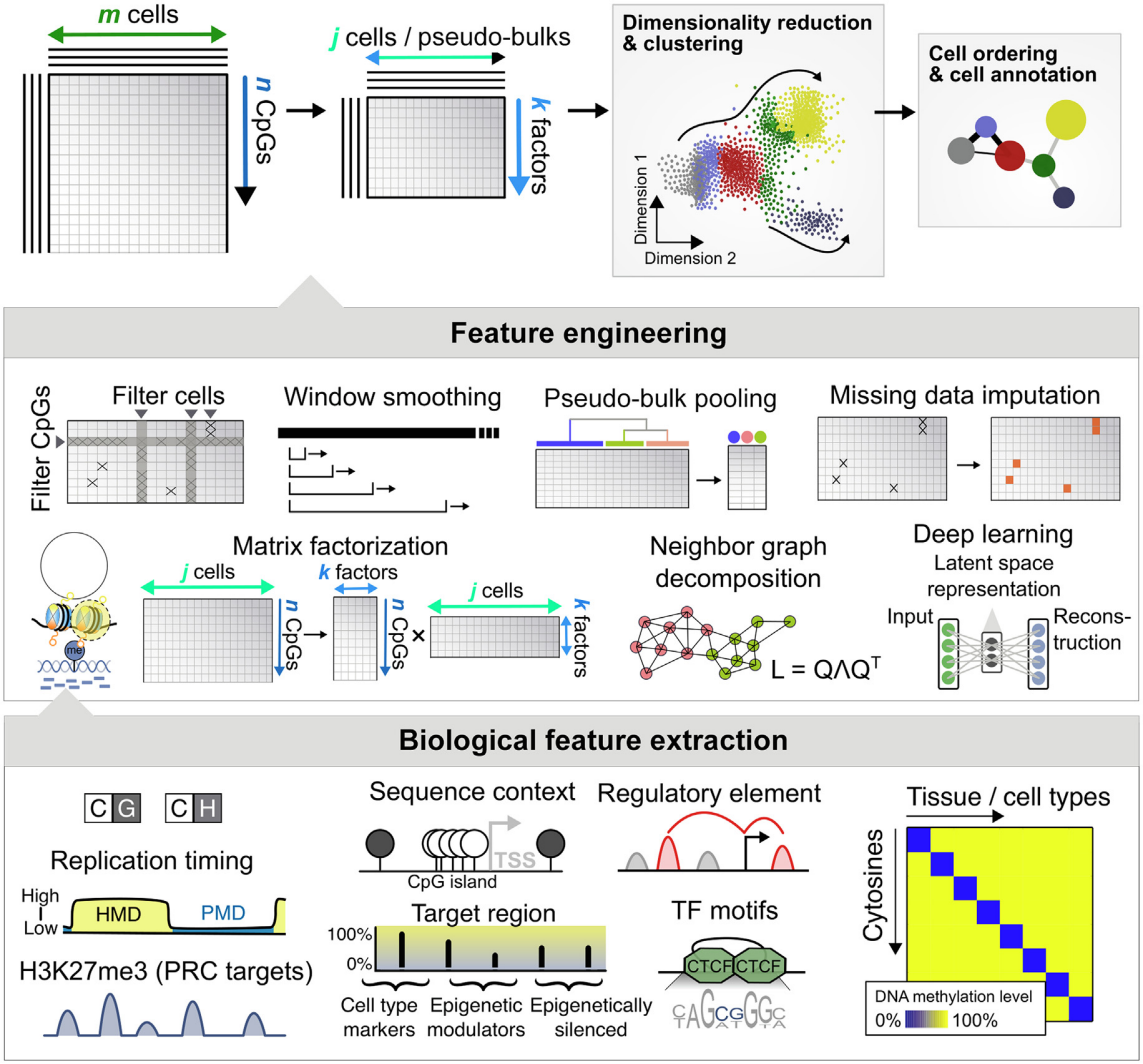
**Schematic representation of data-driven and biological feature selections** After quality control and data preprocessing, the major components of most bioinformatics tools are feature engineering (middle panel) and biological feature extraction (bottom panel), which are performed before downstream analyses such as cell clustering, annotation, and trajectory analysis. Feature engineering: (1) filtering CpGs and samples (based on read depth, data sparsity, and variance); (2) window smoothing (based on defined window sizes and step sizes); (3) pseudo-bulk creation; (4) imputation of missing values; (5) matrix factorization; (6) neighbor graph decomposition; and (7) deep learning latent space representation. Biological feature extraction: (1) methylation extraction from CpG and CpH (where H represents A, C, or T), respectively; (2) identifying replication timings based on CpG methylations in HMDs and PMDs; (3) DNA methylation on PRC targets with histone H3K27me3 marks; (4) sequence context-specific methylations [an example describing unmethylated CpG islands (black and white circles denote methylated and unmethylated cytosines, respectively) at specific gene promoters]; (5) methylations in regulatory element sites and TF binding motifs (an example showing CCCTC-binding factor, CTCF, and binding); and (6) cell type- and tissue-specific methylation signatures. HMD, highly methylated domain; PMD, partially methylated domain; PRC, polycomb repressive complex; TSS, transcription start site; TF, transcription factor.

Data sparsity is a defining challenge common to all single-cell data [124], [125], [126]. This challenge is exacerbated in single-cell DNA methylomes due to the limited DNA material per cell [88]. For scRNA-seq, scATAC-seq, and scHiC data, biological and nonbiological zeros are hard to discriminate [127], [128]. Single-cell methylome signal is based on chemical conversion and is uniquely exempt from having this problem. For single-cell methylome data, missing data are explicitly reflected on the read depth, attributable to the dropout of one or both alleles. These two scenarios can be hard to discriminate when one allele is lost and the other gets amplified in the experiment. The dropout of one allele could lead to misinterpretation of the methylation signal at mono-allelically methylated sites. Some downstream analyses may require a complete data matrix without missing values. Here we discuss three common strategies to address the data sparsity challenge (Figure 3).

#### Cell and feature filtering

Filtering cells with suboptimal genome coverage or mapped read counts can effectively reduce data sparsity (Figure 3). However, single-cell methylome often has distinct technical characteristics and targets different biology; therefore, different thresholds need to be applied for filtering low-quality data. For example, Hernando-Herraez et al. discarded cells with less than 1,000,000 reads or 500,000 CpGs covered and down-sampled each cell to 1,000,000 reads per cell to avoid sequencing depth bias [129]. Liu et al. filtered cells with less than 500,000 reads [66]. Gaiti et al. filtered cells with less than 50,000 CpGs covered using scRRBS [68]. In addition, one can also exclude genomic regions sparsely represented in the data and regions flagged for other quality issues [130].

#### Cell and feature aggregation

To further enhance the signals from sparse data, one could aggregate signals from multiple CpGs to reduce data dimensionality and alleviate data sparsity without explicitly recovering the missing data for each CpG. Genome tiling or window smoothing is a simple but effective method (Figure 3). Methylation fraction averaging can be done with sliding windows of different lengths and step sizes. However, the choice of the window size is often constrained by cost and experimental limitations. Windows range from 1 kb-size non-overlapping windows [100], 3 kb-size 600 bp-stepping windows [61], to 100 kb-size non-overlapping windows [63], [66]. Choice of window size affects the quantification of cell-to-cell similarity. For example, large window size of 100 kb may dilute focal lineage-specific enhancer signals and diminish cell lineage discrimination. Alternatively, one can perform analyses based on shared genomic and epigenomic features where methylation readings are aggregated and cells are compared on genome-wide aggregates [74], [100]. Most targeted genomic features are gene-centric. They include gene promoters, gene body with flanking regions [66], proximal and distal regulatory elements (from, *e.g.*, FANTOM5 [131], the Ensembl regulatory build [132], and ENCODE [133]), and accessible chromatin identified from bulk and scATAC-seq [96]. Genomic features can also be extracted from data using unsupervised methods such as by maximizing methylation variation across cell samples [96], Variational Bayes [134], or models based on the curated annotation of epigenetic markers [63].

Besides feature aggregation, one could aggregate signals from similar cells and produce a single pseudo-bulk methylome to represent a cell group (Figure 3). The underlying rationale of this approach is that when cells from the same group are sufficiently similar, it is safe enough to ignore the residual heterogeneity in exchange for higher genome coverage. The subsequent analysis focuses on analyzing the pseudo-bulk profiles rather than individual single cells [63], [64], [66], [72]. One may also build different pseudo-bulk samples to mimic sample replicates for analysis that requires replicates. This pseudo-bulk pooling can be done by sampling cells with or without overlaps [120]. Accurate clustering of cells with similar epigenomes is key to data aggregation with minimum information loss.

#### Data imputation

Several data imputation methods have also been proposed to handle missing bulk and single-cell DNA methylome data (Figure 3). Zhang et al. proposed a random forest method to impute missing DNA methylation data from bulk methylome data using neighboring CpG methylation levels and epigenomic annotations [135]. BoostMe used XGBoost, a gradient boosting algorithm, for imputing low-quality WGBS data [136]. Farlik et al. imputed missing CpG methylations using the impute R package with 5-nearest neighbor averaging [137]. Luo et al. [63] and EpiScanpy [138] imputed CpH methylations by replacing the missing bin values with the average methylation across all cells for those bins. DeepCpG used a convolution neural network (CNN) framework to impute DNA methylation levels in single cells [139]. MethylStar is a pipeline that wraps in METHimpute, a hidden Markov model-based method [140], for single-cell methylome analysis [141]. Hui et al. used Bsmooth [142] to estimate methylation levels at all CpG sites in the genome [62]. Melissa [143] and Epiclomal [144] used Bayesian mixture models that jointly cluster sparse single-cell methylomes and impute missing values. LightCpG [145] and CaMelia [146] both used gradient boosting to train classifiers with reduced training time and increased accuracy, leveraging positional, structural information, and other bulk methylome data. CpG Transformer adapted the transformer neural network architecture to allow parallel imputation and better scaling on large datasets [147].

### Non-CpG methylation

Although cytosine methylations predominantly occur at CpG dinucleotides, they can also be found at non-CpG (referred to as CpH or CH) sites in specific tissues and cell types [109] (Figure 3). Non-CpG methylations primarily occur at CpA sites [106] in embryonic stem cells (ESCs) [37], neurons [63], and oocytes [148]. While gene activity is associated with CpG methylation at gene promoters and enhancers, non-CpG methylation over gene bodies was found to be more predictive of gene expression in neurons [149]. It can negatively correlate with transcriptional activity in single-cell methylomes [63], [66]. Non-CpG cytosines can be classified into CHG and CHH cytosines [148], whose methylation is usually highly correlated in mammalian cells [106].

### Data techniques customized for single-cell methylome analysis

#### Manifold learning and dimensionality reduction

DNA methylation data have a higher dimension than gene expression data (∼ 28,000,000 CpGs *vs.* ∼ 20,000 genes for human genome). To understand cell-to-cell similarities and facilitate data visualization, one can perform dimensionality reduction techniques that project cells from the original data space into a lower-dimensional (*e.g.*, 2D) feature space (Figure 3). One can also partition the whole-cell population into similar sub-populations using clustering analysis. These two analyses are closely related, and both rely on learning the cell-to-cell distances (or similarities) in the original data manifold.

Besides direct feature aggregation, most dimensionality reduction techniques are based on matrix factorization, neighbor graph decomposition, and generative model inference. Matrix factorization methods can be classified into linear methods such as principal component analysis (PCA) [150], non-negative matrix factorization (NMF) [151], and nonlinear methods such as multi-dimensional scaling (MDS) [152]. Notable examples of neighbor graph-based methods are t-distributed stochastic neighbor embedding (tSNE) [153] and uniform manifold approximation and projection (UMAP) [154]. Generative model-based methods include Bayesian nonparametric models [155] and deep generative models. These methods are often used in combinations to avoid the dominance of data sparsity in downstream analyses and reduce computational load. For example, Farlik et al. first aggregated methylations using 1 kb tiles and then applied MDS to project each cell’s methylome to 2D [100]. The same team also applied PCA to methylation aggregation according to TF binding sites in a later study [137]. Luo et al. first aggregated methylations using 100 kb tiles before applying tSNE [63]. Hui et al. performed MDS using a global dissimilarity measure that averages the absolute methylation difference [62]. Mulqueen et al. first performed NMF to extract latent variables before using tSNE to project the data to the 2D space [64]. MOFA and MOFA+ adapted a group factor analysis framework and applied mean-field Variational Bayes inference to factor matrices with single-cell data from multiple modalities, including DNA methylomes [156]. The same authors co-opted MOFA for data visualization by restraining the number of latent variables to 2 [96]. Liu et al. first grouped cells on three hierarchical levels, *i.e.*, cell class, major type, and subtype, and applied UMAP and tSNE on each level [66].

#### Clustering

Cell clustering [157] often serves as a primary step in analyzing cell population structures [48]. The resulting cell clusters can be used in data imputation, pseudo-bulk methylome construction [66], trajectory inference [158], and cell type refinement (Figure 3). Clustering methods used in previous single-cell DNA methylome studies include k-mean clustering [159], k-medoid clustering [160], density-based clustering (*e.g.*, mean-shift [161] and density-based spatial clustering of applications with noise (DBSCAN) [162]), hierarchical clustering [163], nonparametric Bayesian methods (*e.g.*, Dirichlet mixture models [164]), affinity propagation [165], and neighbor graph-based clustering (*e.g.*, community detection [166], [167], spectral clustering [168], modularity [169], Louvain clustering [170], Infomap [171], and Leiden clustering [172]). Most clustering algorithms share their mathematical roots with dimensionality reduction methods. Choosing the optimal clustering method depends on the geometric shape of the expected clusters and practical computational constraints; see [48] for a detailed discussion. There are many direct adaptations of the standard clustering methods for single-cell methylome data analysis. Of these methods, hierarchical clustering is one of the most widely used [65], [72], [74], [137] and adapted (*e.g.*, by BackSPIN [173] and PDclust [62]). Mulqueen et al. used DBSCAN after dimensionality reduction [64]. Liu et al. used the Leiden algorithm for clustering [66]. Melissa [143] and Epiclomal [144] used probabilistic mixture models to assign cells to clusters while simultaneously imputing missing data. Epiclomal employed a Bernoulli mixture model [144] but used density-based clustering and hierarchical clustering methods for model initialization. To reconcile different clustering results or accommodate randomness in the clustering algorithms, consensus clustering can be used on top of multiple clustering assignment matrices [174], which might be derived from running multiple clustering algorithms or the same stochastic clustering algorithm multiple times. Liu et al. treated each clustering assignment from multiple Leiden clustering runs as the new feature for each cell and performed DBSCAN to achieve consensus clustering [66].

#### Cell ordering and lineage reconstruction

To capture the continuity of cell state evolution, one often orders cells or cell groups to quantify cell state transitions such as differentiation and cell cycle changes (Figure 3). Here we will use the term of cell ordering (total or partial order). The readers may find similar analyses referred to as pseudo-time ordering, trajectory inference, lineage reconstruction, and taxonomy reconstruction in the literature. Methods are available to accommodate different trajectory topologies ranging from linear, bifurcating, multifurcating, tree-like to acyclic and cyclic graphs (see Saelens et al. [175] for a survey and benchmarking).

Strategies used in previous single-cell methylome analyses include visual inspection, neighbor graph-based methods, and model-based methods. Farlik et al. studied the role of methylation in lineage differentiation of mouse ESCs by comparing 5-azacytidine-treated and untreated cells [100]. 5-azacytidine causes a global decrease in methylation due to its inhibition of DNA methyltransferases (DNMTs). Cells are ordered in a lineage plot spanned by the positive and negative methylation differences. EpiScanpy [138] uses PAGA [158] for cell ordering. PAGA was initially developed to infer trajectory from scRNA-seq data. It first constructs a k-nearest neighbor-like graph within UMAP and then partitions the graph using the Louvain method. Finally, a new abstraction graph is constructed, treating cell groups as nodes in the new graph. The connectivity between nodes in the new graph quantifies the ratio of observed over expected number of inter-group edges in the original graph [158]. Clark et al. used the diffusion pseudo-time (DPT) method [176] to infer developmental trajectory from the gene expression component of scNMT-seq data [77]. DPT constructs a weighted neighbor graph before performing a random walk to model cell state transitions. Popular alternative approaches to infer trajectory from neighbor graphs include the use of a minimum spanning tree (*e.g.*, in Monocle [177]) and the shortest path algorithms (*e.g.*, in Wanderlust [178] and Wishbone [179]). These methods remain to be tested on single-cell DNA methylome data.

Besides neighbor graph-based methods, one can also use model-based statistical inference methods, *e.g.*, phylogenetic reconstruction techniques, to infer lineage relationships among cells. Unlike neighbor graph-based methods, phylogenetic methods assume that observed cells are at the leaves of the phylogeny (akin to extant species), and the methylation states of the internal nodes will be inferred. To account for DNA methylation heterogeneity at different genomic regions, Gaiti et al. [68] first selected a base-substitution model using a model-selection procedure that incorporates rate heterogeneity across sites [180]. The authors then applied IQ-TREE [181], a maximum likelihood-based phylogenetic tree searching algorithm, in which branch support is estimated using the ultrafast bootstrap (UFBoot) method [182] combined with a Shimodaira-Hasegawa-like approximate likelihood ratio test and an approximate Bayes test [68]. The robustness of the tree construction is further evaluated by holding out specific chromosomes or CpGs [68]. This maximum likelihood-based approach is over 3-fold more robust than maximum parsimony-based reconstruction. Furthermore, the branch lengths (the cumulative methylation change) can be further translated to the number of cell divisions using rates previously calibrated in colorectal cancer [183].

#### Supervised cell annotation

DNA methylome-based cell type annotation often leverages data of known cell types and markers (CpGs or genes) with known cell type associations (Figure 3). Farlik et al. applied an elastic net-regularized generalized linear model [184] with training data labels derived from data pooled from cells of known identity [137]. Model features were extracted from regulatory regions defined in the BLUEPRINT Ensembl regulatory build. Luo et al. [63] and Liu et al. [66] annotated neuronal and other brain cells using global CpH methylation and locus-specific methylation at marker genes with known methylation–expression correlation. Luo et al. first clustered cells using the BackSPIN method [173] and annotated clusters based on the depletion of CpH methylation at genes (whole gene bodies ± 2 kb) whose expression levels are known to mark neuron subtypes [63]. Liu et al. first classified cells at three hierarchical levels and then carried out cell type annotations within levels to maximize the power of subtype discrimination [66]. Aside from cell type information, one can also annotate other aspects of the cell state. For example, Guo et al. annotated cell ploidy using controlled lambda DNA spike-in [76]. Hernando-Herraez et al. annotated the epigenetic age of single-cell methylomes using a linear model fitted on bulk-tissue data [129]. Recently Trapp et al. introduced a percentile-based approach to address coverage discrepancy from single cells to track the cellular aging process using single-cell methylomes [185]. Johnson et al. [186] annotated the tumor type from scRRBS data using the MolecularNeuropathology classification tool [187].

#### Differential methylation modeling and motif analysis

**Figure 4:**
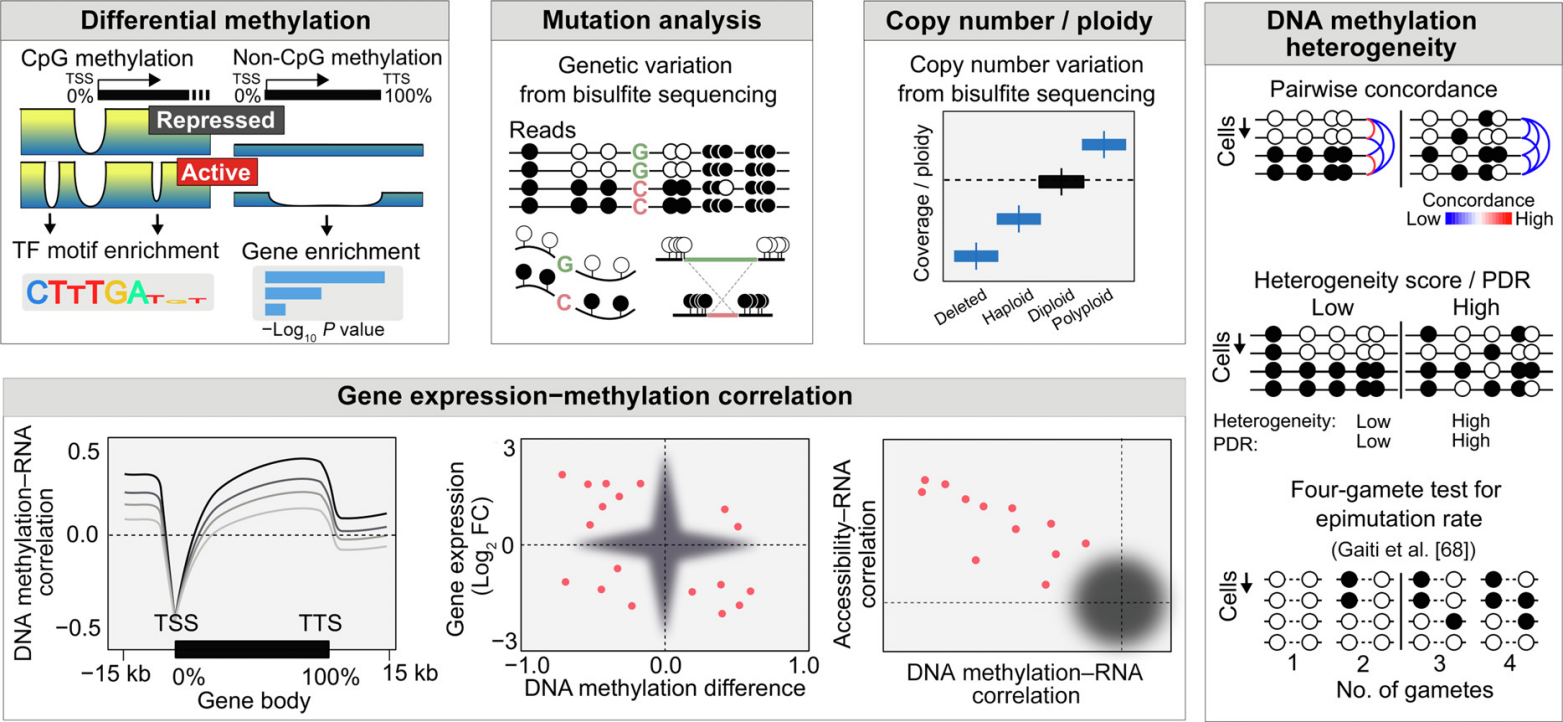
**Schematic representation of DNA methylation variation and its association with mutational, transcriptional, and chromatin accessibility signals** Differential methylation: (1) CpG methylation differences at regulatory elements (these differences can be associated with TF binding and can be enriched for binding motifs); (2) non-CpG (CpH) methylation differences at gene bodies (these differences can predict gene transcriptional states and can be used for pathway enrichment analysis). Mutation analysis: mutation calling from bisulfite sequencing (white circles represent unmethylated reads, and black circles represent methylated reads). Copy number / ploidy: genome coverage signal can be used to determine ploidy using bisulfite sequencing. Gene expression−methylation correlation: (1) correlation of DNA methylation with RNA expression varies across genomic regions (*e.g.*, TSS methylations may be anti-correlated or uncorrelated with RNA expression, while gene body methylations may be positively correlated with RNA expression); (2) comparison of RNA expression change with DNA methylation change in single cells; (3) comparison of correlations between DNA methylation and RNA expression and between chromatin accessibility and RNA expression. Open accessibility regions are positively correlated with gene expression, while DNA methylation (*e.g.*, at enhancers) is generally negatively correlated with gene expression. The pink dots represent outlier samples profiled for RNA expression and DNA methylation (middle panel) or RNA expression, chromatin accessibility, and DNA methylation (right panel). DNA methylation heterogeneity: (1) pairwise concordance of cells (high concordances are marked by red lines, and low concordances are marked by blue lines) can be determined based on methylation patterns (white circles indicate unmethylated cytosines and black circles represent methylated cytosines); (2) methylation states (white circles indicate unmethylated cytosines and black circles represent methylated cytosines) in cells are used for heterogeneity scoring based on PDR; (3) determining epimutation rate using the four-gamete test (figure adapted from Gaiti et al*.*[68]). TTS, transcription termination site; PDR, proportion of discordant reads.

DNA methylation differences across cells, cell clusters, and genomic regions can be inferred using standard statistical tests or methods previously developed for bulk methylome analyses (Figure 4). Statistical significance, effect size, and multiple test correction are three commonly used filtering criteria in calling differentially methylated CpGs (DMCs), differentially methylated windows (DMWs), differentially methylated CpG islands (DM-CGIs), and differentially methylated regions (DMRs). For example, Gravina et al. used two-sided *z*-tests and *t*-tests for DMW detection [61]. Hui et al. first calculated DMCs using the z-score method before merging them into DMRs [62]. Hou et al. used Fisher’s exact test for detecting DM-CGIs and required *P* < 0.05 and a minimum methylation difference > 0.3 between two subpopulations [72]. Farlik et al. first identified consistently methylated regions and then used a *t*-test with a *P* value cutoff of 0.01 to assess whether a region is a DMR [100]. Zhu et al. [123] and Bian et al. [73] used a false discovery rate (FDR)-adjusted Student’s *t*-test to detect 300 bp DMWs with FDR > 0.05 and polarized methylation levels ≥ 0.8 in the higher group and ≤ 0.2 in the lower group. Gaiti et al. defined DMRs based on the absolute weighted methylation difference (> 0.3) and a two-sided nonparametric permutation test (*P* < 0.05) [68]. Luo et al. [63] and Liu et al. [66] used the DMRfind function in methylpy [109] to calculate DMRs across subtypes. Luo et al. further merged neighboring DMRs into larger DMRs at least 1 kb apart. To detect regions that lack methylation and are potentially subject to regulatory machinery binding, Li et al. used MethylSeekR to call lowly methylated regions (LMRs) in human ESCs [188]. scMET models the number of methylated CpGs using a beta-binomial model before using a generalized linear model framework to test differential methylation [189]. This framework explicitly accounts for sequence feature-specific factors (*e.g.*, CpG density) to disentangle overdispersion of biological causes from technical variations.

DMCs, DMRs, and LMRs may be further scanned for the presence/enrichment of sequence motifs using tools such as hypergeometric optimization of motif enrichment (HOMER) [190] or the multiple expectation maximizations for motif elicitation (MEME) suite [191]. The identified motifs can then be matched against TF binding motifs in well-curated databases based on motif–motif similarity (Tomtom [192]). Commonly used motif databases include TRANSFAC [193], UniPROBE [194], JASPAR [195], Cis-BP [196], TFClass [197], and HOCOMOCO [198]. For testing enrichment, the choice of background is critical. For example, analyses testing for the enrichment of TF binding motifs often use H3K27ac sites as the background [199] to look for differential methylations associated with enhancer regions. H3K27ac peaks are generally associated with all transcriptionally activated TF bindings and can be used as the null distribution when testing for the binding of specific TFs.

## Mutation and copy number analysis

Like many omics data, single-cell methylome data harbor rich information regarding the DNA itself, such as genetic variation [200] and copy number alterations [201] (Figure 4). Zhu et al. used a binomial test and Bis-SNP [99] to call SNPs from bisulfite sequencing data to identify parental-specific methylations [123]. Similarly, Li et al. identified parental SNPs from scCOOL-seq data and linked neighboring genomic sites to identify parental allele-specific methylations and chromatin accessibility [188]. Farlik et al. compared the methylome coverage of HL60 and K562 cells against HL60- and K562-specific copy number alterations to verify cell identity [100]. Hou et al. [72] inferred copy numbers from the scTrio-seq data by using a hidden Markov model (HMM) [202]. Guo et al. used HMMcopy [203] to infer copy numbers using normalized read counts from scCOOL-seq data [76]. Bian et al. [73] and Johnson et al. [186] used Ginkgo [204] to infer copy number alterations from single-cell methylomes.

## Stochastic DNA methylation variation and heterogeneity

Measuring DNA methylome at the single-cell resolution allows us to distinguish stochastic methylation changes (epigenetic drift) from coordinated methylation changes by studying consistency in the local methylation patterns within and across cells (Figure 4). With bulk DNA methylome data, this was done mainly through read-level analyses using metrics such as epi-polymorphisms [205], proportion of discordantly methylated reads (PDR) [206], methylation entropy, and methylated haplotype load (MHL) [207]. Smallwood et al. computed the cell-to-cell variance in methylation from single-cell methylomes and found that CpGs associated with active enhancer elements have significantly higher variances of methylation than CpGs in CGIs and intracisternal A-particle repeat DNA [65]. Farlik et al. tracked the pairwise Euclidean distance among single cells before and after 5-azacytidine and vitamin D treatment. They identified a temporary surge in variability as cells individually transition in response to treatment [100]. Hernando-Herraez et al. developed a normalized methylation heterogeneity score to detect the hallmarks of aging and to identify DNA regions influenced by epigenetic drift [129], [208]. The score is based on Hamming distance and Shannon entropy and accounts for the dependency of methylation variances on the methylation means [129]. Gaiti et al. measured epimutation rates based on the four-gamete test that allows for the calculation of methylation heterogeneity in CpG-sparse regions [68]. Johnson et al. used the PDR to conclude that glioma cells have a higher epigenetic heterogeneity in comparison to normal cells [186].

## Integration with scRNA-seq data in different sample spaces

To facilitate the integration of DNA methylation with gene expression data from different sample spaces, one can leverage cytosine methylations correlated with gene expression to predict gene transcriptional activity in a computational procedure sometimes referred to as gene activity scoring [134], [209], [210], [211]. Despite the extensive study of DNA methylation as a regulator of gene expression [5], [6], [7], [212], it is often nontrivial to precisely characterize the methylation–expression relationship, which often depends on the cell type and genomic context of the cytosines. For example, although promoter CpG methylations were thought to be negatively associated with the gene expression, it is no longer considered a general genome-wide rule for most genes. Farlik et al. found that only a small number of differentially expressed genes showed differences in methylation [137]. This is in part due to the orthogonal gene expression regulatory process, *e.g.*, silencing via the polycomb repressive complexes, and other methylome-impinging processes, such as the cell division effect on late replicating DNA. In fact, 70%−80% of genomic CpGs were estimated to have stable methylation states, and only ∼ 15%−21% have dynamic methylation patterns in adults, depending on the tissue types [109], [213]. As such, feature selection can be critical to the success of predicting gene expression based on DNA methylation. Luo et al. [63] and Liu et al. [66] leveraged the association between gene body non-CpG cytosine methylations and gene expression activity to find methylation markers for neuron subtypes. However, this approach can be limited to a subset of cells with high non-CpG cytosine methylations, such as neurons [109]. CpG methylations at *cis*-regulatory elements are more commonly used for other cell types to inform gene expression regulation [18], [137].

**Figure 5:**
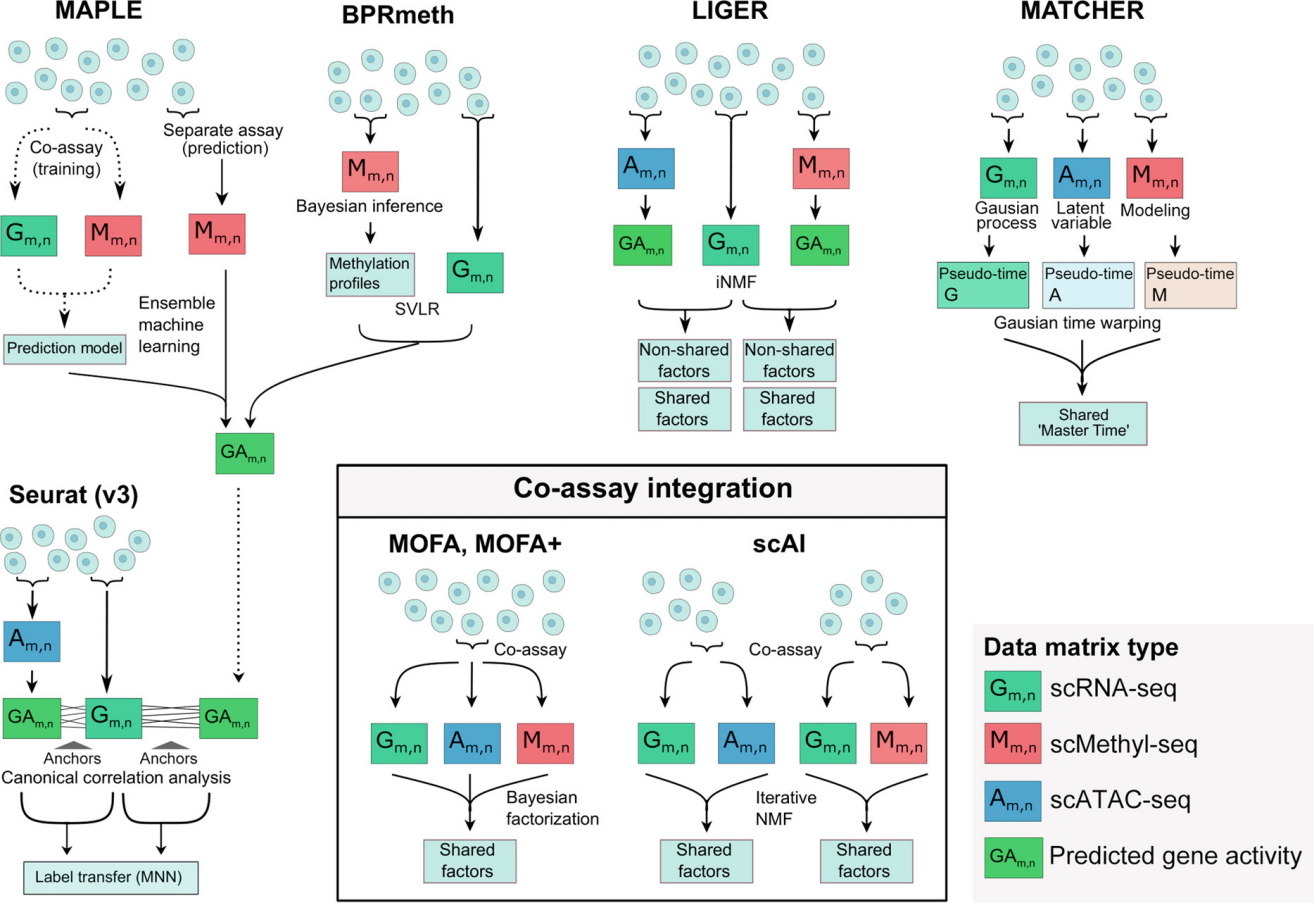
**Overview of bioinformatics tools that integrate single-cell methylome data with other omics datasets** The top panel includes the bioinformatics tools that can incorporate data from different cells for multi-omics integration. MATCHER projects each data modality to a pseudo-time scale. Multi-omics integration can also be achieved through integrating scRNA-seq data (as by LIGER and Seurat) with gene activity matrix predicted from scATAC-seq and scMethyl-seq data (as by LIGER, BPRmeth, and MAPLE). In Seurat, ‘anchors’ between datasets are identified using canonical correlation analysis followed by label transfer using the MNN method. In LIGER, iNMF identifies shared and non-shared factors between datasets. BPRmeth first learns methylation features or profiles of a given cell type and then uses SVLR to predict gene expression corresponding to the methylation profiles. MAPLE produces the gene activity matrix by ensemble machine learning. MOFA and scAI integrate scMethyl-seq data with the other modalities co-assayed in the same cells in the bottom panel. scRNA-seq, single-cell RNA sequencing; scMethyl-seq, single-cell methylation sequencing; scATAC-seq, single-cell assay for transposase-accessible chromatin sequencing; MNN, mutual nearest neighbor; NMF, non-negative matrix factorization; iNMF, integrative non-negative matrix factorization; SVLR, support vector linear regression; scAI, single-cell aggregation and integration.

Several strategies have been adopted to predict gene expression from DNA methylation, including using support vector linear regression (SVLR) by BPRmeth [134] and ensemble machine learning by MAPLE [211], which leverage previously generated co-assay data as training [74], [77], [96], [129], [189] (Figure 5). These methods are designed to be used in combination with specific feature engineering methods such as fixed-size window smoothing [211] and Bayesian clustering [134]. The predicted synthetic gene expression matrices can then be co-analyzed with single-cell gene expression datasets using canonical correlation analysis (*e.g.*, by Seurat [214]), mutual nearest neighbor (MNN) analysis (*e.g.*, by Scanorama [215]), weighted nearest neighbor analysis (WNN) (*e.g.*, by Seurat [216]), and the batch-balanced k-nearest neighbor method (BBKNN [217]). For example, EpiScanpy employed BBKNN to co-embed data from different sources [138].

Alternatively, one can also integrate DNA methylation and RNA-seq data without explicit gene expression prediction (Figure 5). MATCHER projects cells from both the DNA methylation and RNA-seq datasets to a “master” pseudo-time scale (0 to 1) using a Gaussian process latent variable model (GLVM) [176]. The two projections can then be combined to establish cell-to-cell correspondence through manifold alignment [218]. One can also perform joint matrix factorization using this alignment (*e.g.*, by coupled NMF [219], LIGER [210], CSMF [220]). Notably, LIGER [221] and its online adaptation [222] employ integrative NMF (iNMF) [223] for joint dimensionality reduction of single-cell expression and methylome data. Cells can then be represented on a shared factor neighborhood---a low-dimensional space spanned by factor loadings. This analysis can be semi-supervised by biologically-guided anchor cells and anchoring features (see reviews of integrating multiple omics datasets [46], [224] for the principle of this approach).

## Multi-omics co-assays including DNA methylome

Single-cell multi-omics co-assay technologies profile multiple omics data in the same cells and bypass the challenge of aligning cells from single-cell methylomes to other data modalities [225]. Notable single-cell co-assay methods include ones that combine DNA methylation with gene transcription (scM&T-seq [74], scTrio-seq [72], and smart-RRBS [70]), chromatin accessibility (scNOMe-seq [75] and ATAC-Me [226]), both gene transcription and chromatin accessibility (scCOOL-seq [76] and snNMT-seq [77]), and 3D genome conformation (methyl-HiC [79] and sn-methyl-3C [80]). One can study the relationship of DNA methylation with other molecular phenotypes directly in the same cells. Hou et al. [72] and Bian et al. [73] verified the known negative association of gene expression with promoter methylations and positive association with gene body methylations in single cells using scTrio-seq. Gaiti et al. used Smart-RRBS and identified a negative correlation between gene expression and promoter DNA methylations in normal B cells and more prominently in lymphocytic leukemic cells [68]. Applying scNMT-seq to mouse gastrulation cells, Argelaguet et al. identified 125 genes with expression significantly correlated with promoter methylations [96]. Based on a Bayesian group factor analysis framework, the authors also developed and applied MOFA [156] and MOFA+ [227] to perform dimensionality reduction and integration in the reduced latent factor space (Figure 5). Similarly, scAI also took a unified matrix factorization approach to analyze single-cell multi-omics co-assay data that included the DNA methylome [228] (Figure 5).

## Ongoing challenges and future directions

Many methods have been developed for analyzing scRNA-seq and scATAC-seq data. However, single-cell DNA methylome analysis methods remain relatively limited, with comprehensive software tool suites only emerging recently [138], [229]. This is partly attributable to the DNA methylome being high in dimensionality and low in copy number. The probability of missing DNA methylation information per CpG is higher than that of missing a transcriptional signal in a scRNA-seq experiment of similar sequencing depth. As a result, compared to scRNA-seq data, analyzing single-cell DNA methylome data is often more challenging and demands more advanced feature selection.

The challenge of single-cell DNA methylome analysis can also be attributed to the complex grammar of DNA methylation determination, coordinated by the biochemical and cellular processes that deposit, dilute, and erase the methylation marks on the cytosine bases. First, these processes often operate at different genomic scales. For example, replication timing-associated DNA methylation change takes place on mega-base-pair-scale domains [42], [43], [230]. Non-CpG cytosine methylations are correlated with gene transcription at gene bodies in neurons [66]. TF binding dictates more focal DNA methylation patterns [231], [232]. The difference in these scales of representation requires feature selection and analysis to be performed at different genomic scales [44]. Second, supervised analysis of focal lineage-specific or disease-specific methylation alterations needs to account for processes that impact the DNA methylation level globally to avoid confounding effects. These processes include the action of methylation readers, writers, and other cellular phenotypes, such as the cell cycle stage, which is not usually included in the bulk methylome analysis. Supervised single-cell annotation methodology based on grammar learned from bulk DNA methylomes is yet to be developed.

An implication of the multi-factor determination of DNA methylation is the relative lack of data that capitulate single-cell DNA methylome under diverse biological conditions. Although we are witnessing an explosive increase in the single-cell DNA methylome data volume [88], most data are assayed in individuals of one age, one genetic background, and one pathological state. For example, state-of-the-art tools like LIGER [210] and MAPLE [211] were trained using a limited number of real datasets and synthetic datasets; therefore, whether their performance can be extended to other biological and technical scenarios remains to be verified. Nevertheless, several ongoing single-cell multi-omics data consortia have started to include DNA methylation and will generate more detailed DNA methylome references under more diverse conditions  [233].

Aside from the complex biological grammar, current public single-cell methylome data are often obtained using different assay technologies [53], which challenges their integration. For example, combinatorial barcoding usually yields more cells at lower sequencing depth and is more susceptible to allelic dropout than plate-based methods [64], [234]. The discrepancy of different assay technologies in genomic coverage can also cause systematic bias when the window-smoothed signal is compared. Whether solutions to correct batch and platform-specific effects in scRNA-seq data [235] can be applied to DNA methylome data is yet to be validated. The discrepancies among assay technologies also require more general and customizable computational methods. A comprehensive benchmarking of single-cell methylome assay technologies and analysis tools (like existing benchmark efforts for the scRNA-seq data [127], [175]) is a pressing unmet need. Methods that can adequately integrate data from different assay technologies and provide a standard cell-specific methylome reference need to be better developed.

The prevalence of single-cell transcriptome and chromatin accessibility data [236], [237], [238], [239] presents the question of how DNA methylome data can be co-analyzed with scRNA-seq and chromatin accessibility. The added value provided by single-cell DNA methylome data needs to be better quantified. Multiple methods have been proposed to analyze single-cell methylome data and scRNA-seq data in different [210], [211] and same sample spaces [156], [227], [228]. Besides integration with scRNA-seq data, integration with scATAC-seq data is also feasible. For example, Li et al. used scCOOL-seq to report a negative correlation between chromatin accessibility and DNA methylation in embryonic cells [188]. Integration with single-cell chromatin accessibility data remains an active direction of development. The coupleCoC+ tool uses an information-theoretic co-clustering framework for integrating multimodal single-cell genomic datasets, including single-cell methylome data [240].

The rich resource of informatics tools for scRNA-seq analysis [241] also raises the question of whether these tools can be effectively applied to single-cell DNA methylome data. Many scRNA-seq analysis strategies, *e.g.*, lineage construction, share the same principle as DNA methylome analyses. Therefore, repurposing these scRNA-seq tools for DNA methylome analyses can be highly feasible. For example, Liu et al. [66] extensively used Scanpy [242] for selecting variable methylations, dimensionality reduction, and nearest neighbor graph construction. EpiScanpy [138] used PAGA [158] for lineage inference and cell type abstraction. Complete repurposing of other tools for single-cell DNA methylome data requires coordinated efforts from experts in different omics fields.

## Conclusion

The increasingly widespread use of single-cell DNA methylome profiling for tracking cell identity and understanding gene regulation in biomedical research and clinical applications has been producing single-cell methylome data on scales of increasing magnitude. The increase in the volume of single-cell methylome data demands the development of highly efficient, flexible, versatile, and easy-to-use computational tools for its analysis. Advances in analytical tools can help precipitate the adoption of modern high-throughput single-cell DNA methylome profiling technologies. This focus on single-cell methylome profiling will unveil the complexity of intercellular interactions and gene regulation heterogeneity in broad biological and translational contexts.

## Competing interests

Neither of the authors has any competing interest to declare.

## CRediT authorship contribution statement

**Waleed Iqbal:** Writing – original draft, Writing – review & editing, Visualization. **Wanding Zhou:** Conceptualization, Writing – original draft, Writing – review & editing, Visualization. Both authors have read and approved the final manuscript.
